# Lung nodule pre-diagnosis and insertion path planning for chest CT images

**DOI:** 10.1186/s12880-023-00973-z

**Published:** 2023-02-03

**Authors:** Rong-Li Xie, Yao Wang, Yan-Na Zhao, Jun Zhang, Guang-Biao Chen, Jian Fei, Zhuang Fu

**Affiliations:** 1 Department of General Surgery, Ruijin Hospital, Shanghai Jiao Tong University School of Medicine, Shanghai, 200025 China; 2 State Key Laboratory of Mechanical System and Vibration, Shanghai Jiao Tong University, Shanghai, 200240 China; 3 Department of Ultrasound, Tongji Hospital, School of Medicine, Tongji University, Shanghai, 200065 China

**Keywords:** Multiple pulmonary nodules/diagnosis, Diagnostic imaging*, Tomography, X-ray computed, Humans, Lung neoplasms*/pathology

## Abstract

Medical image processing has proven to be effective and feasible for assisting oncologists in diagnosing lung, thyroid, and other cancers, especially at early stage. However, there is no reliable method for the recognition, screening, classification, and detection of nodules, and even deep learning-based methods have limitations. In this study, we mainly explored the automatic pre-diagnosis of lung nodules with the aim of accurately identifying nodules in chest CT images, regardless of the benign and malignant nodules, and the insertion path planning of suspected malignant nodules, used for further diagnosis by robotic-based biopsy puncture. The overall process included lung parenchyma segmentation, classification and pre-diagnosis, 3-D reconstruction and path planning, and experimental verification. First, accurate lung parenchyma segmentation in chest CT images was achieved using digital image processing technologies, such as adaptive gray threshold, connected area labeling, and mathematical morphological boundary repair. Multi-feature weight assignment was then adopted to establish a multi-level classification criterion to complete the classification and pre-diagnosis of pulmonary nodules. Next, 3-D reconstruction of lung regions was performed using voxelization, and on its basis, a feasible local optimal insertion path with an insertion point could be found by avoiding sternums and/or key tissues in terms of the needle-inserting path. Finally, CT images of 900 patients from Lung Image Database Consortium and Image Database Resource Initiative were chosen to verify the validity of pulmonary nodule diagnosis. Our previously designed surgical robotic system and a custom thoracic model were used to validate the effectiveness of the insertion path. This work can not only assist doctors in completing the pre-diagnosis of pulmonary nodules but also provide a reference for clinical biopsy puncture of suspected malignant nodules considered by doctors.

## Introduction

Affected by factors such as smoking, air pollution, and the occupational environment, lung carcinoma is one of the most malignant tumors that poses a threat to human health and life and is the major killer across all cancers [1, 2]. As per the global cancer data, the number of new cases and deaths of lung carcinoma in 2018 were 2.1 and 1.8 million, respectively, and both its morbidity and mortality are among the highest amongst all cancers [3]. The 5-year survival rate of patients with advanced lung carcinoma is only about 16%, but for early patients with effective treatment, the 5-year survival rate can be increased by approximately 4–5 times [4]. Therefore, early diagnosis, screening, and clinical treatment of lung carcinoma are of great significance. Generally, lung carcinoma is characterized by pulmonary nodules at an early stage; however, lung nodules are not necessarily cancer, and most nodules are benign. Therefore, the accurate detection of pulmonary nodules is a prerequisite for reliable clinical treatment.

Usually, pulmonary nodules are < 2 cm in diameter, size > 3 cm is considered a mass, and only a small percentage of small pulmonary nodules may be lung carcinoma or precancerous lesions [5, 6]. With the popularity of imaging techniques such as ultrasound, CT, positron emission tomography (PET), and magnetic resonance imaging (MRI), the detection rate of malignant pulmonary nodules has increased significantly [7]. Although CT or PET-CT examinations scan the patient’s body to form images that can be used to judge benign or malignant nodules, the accuracy is still limited. In clinical practice, the first major method for detecting pulmonary nodules is to obtain a series of medical images of the patient, such as chest CT images, and the doctor judges whether there are nodules, that is, suspected nodules, based on their experience through observation. This process is defined as pre-diagnosis, and involves more time and relies too much on the experience of the doctor for a preliminary judgment of benign and malignant nodules to be made. For uncertain suspected malignant nodules, it is necessary to perform pathological examination of cells in the nodules by puncture and determine the properties of suspected malignant nodules after comprehensive evaluation. Therefore, the use of digital image processing technology to assist doctors in diagnosing can quickly and accurately achieve the segmentation of lung parenchyma and suspected nodules and accomplish the pre-diagnosis of nodules, greatly reducing the doctor’s diagnostic burden. At the same time, an optimized feasible insertion path is then suggested for suspected malignant nodules, which helps to reduce trauma to the patient and the risk of puncture surgery.

It should be noted that numerous published studies have different definitions for detecting pulmonary nodules, most of which are based on machine learning for benign and malignant detection. However, the scope of this work is limited to recognizing whether there are nodules (that is recognizing suspected nodules), aiming to realize the automatic pre-diagnosis of lung nodules, which can be considered a prerequisite procedure for benign and malignant detection. In other words, benign and malignant detections were excluded from the scope. A brief summary of the literature on the description and detection of pulmonary nodules is presented in the following sections.

### Pulmonary nodules

**Fig. 1:**
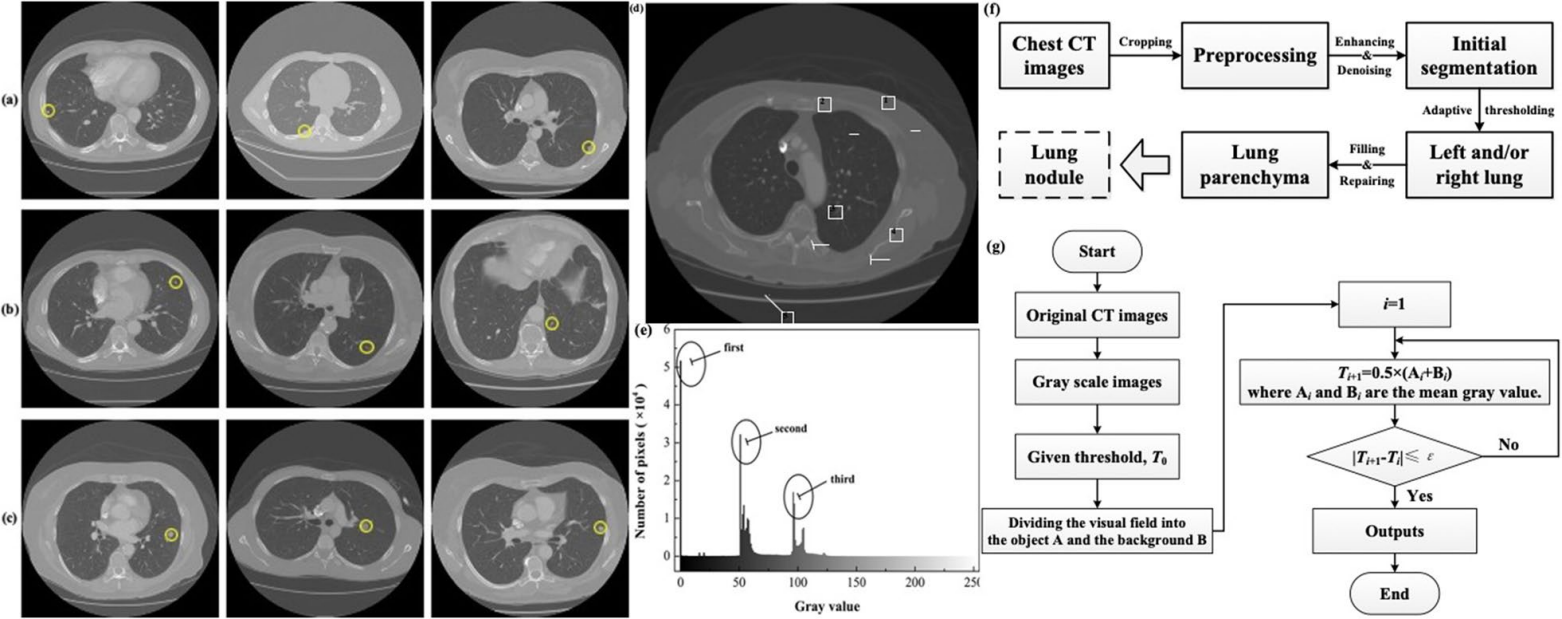
Lung parenchyma segmentation. Typical categories for pulmonary nodules: **a** juxta-pleural pulmonary nodule. **b** Juxta-vascular pulmonary nodule. **c** Solitary pulmonary nodule. An example of chest CT image, **d** original image, **e** gray histogram. The numbers represent as below: 1-outside visual field, 2-air, 3-lung parenchyma, 4-other lung tissues such as muscle, fat, trachea, vessels, etc., and 5-bone. **f** Workflow diagram of lung parenchyma segmentation. **g** Flow chart of preliminary segmentation with adaptive thresholding method

Figure 1a–c show chest CT images of typical pulmonary nodules, which can be divided into three major categories: juxta-pleural, juxta-vascular, and solitary pulmonary nodules, similar to those described in earlier studies [1, 8].

The first two types of pulmonary nodules are often difficult to screen and identify because they adhere to the pleura and blood vessels, respectively. On the other hand, for solitary pulmonary nodules, the diagnosis is relatively easy because of their shape and size, presenting solid or sub-solid opaque spheres. However, it is still a common challenge when the atypical nature of ground-glass nodules or mixed ground-glass nodules with a solid component are encountered [9]. Thus, the detectability of a pulmonary nodule depends mainly on its size, location, attenuation, as well as other characteristics.

### Medical image analysis

Medical imaging technologies have proven to be effective for pulmonary nodule detection and have been regarded as the procedure of choice for screening lung carcinoma. Many imaging devices have evolved depending on the different modalities such as CT, PET and MRI as mentioned above; each having its own pros and cons [10, 11]. For instance, PET can show the metabolic level of the lesion, but the measured standardized uptake value is not absolute because sometimes the metabolism of inflammatory cells can also increase this value leading to misdiagnosis [12]. PET-CT has certain advantages in judging the benign and malignant aspects of small pulmonary nodules [13], but there is controversy in the academic community about the possible harm to the patients due to the amount of radiation exposure. Among these imaging devices, CT is currently one of the most cost-effective methods for pulmonary nodule detection, with high sensitivity and provision of more detailed information about lung disease; the low-dose CT especially can reduce the radiation in patients.

In this study, we primarily focused on the automatic pre-diagnosis of pulmonary nodules. In this regard, the entire imaging process for original chest images typically consists of two steps: lung parenchyma segmentation and suspected nodule classification and diagnosis. Lung parenchyma segmentation is used for nodule detection and is the basis of nodule positioning and percutaneous lung biopsy. It mainly involves initial segmentation and boundary repair. Common methods for the initial segmentation of lung parenchyma include the threshold method, clustering method, and region-growth algorithm. John and Mini [14] proposed an approach for pulmonary nodule segmentation and feature extraction using multilevel thresholding, and their results showed that the proposed method is best suited for the detection of isolated solid nodules. Later, in 2019, Khan [15] used a combination of fuzzy logic and a color feature-based fuzzy C-means clustering method to segment the lung parenchyma. For the boundary repair method, there are the rolling ball method, mathematical morphology method, convex hull, and snake algorithm [16–20]. When the boundary defect is large, the rolling ball method to repair the boundary easily fails, and the convex hull algorithm is also ineffective. Additionally, the snake-algorithm-based repair method is too sensitive to the initial position and easily falls into the local optimum. Lung parenchyma segmentation is not only the key procedure of imaging processing to eliminate impurities or other tissues such as muscle, fat, trachea, sternum, and varied vessels, but also has a great impact on the classification and diagnosis of suspected nodules owing to the complexity and similar intensity of lung structures.

Since the time of AI, in the case of known nodules being labeled, a great deal of research concerning the benign and malignant detection of pulmonary nodules has been reported, and some valuable references can be used for other steps of imaging processing, suspected nodule classification, and diagnosis. However, because of the sparsity and imbalance of medical image databases, existing research on the application of medical image analysis toward the accuracy and efficiency of identification is still a challenge. To the best of our knowledge, detection methods for benign and malignant pulmonary nodules can be divided into two major categories: machine learning-based and rule-based classification methods [21–24]. Machine learning methods such as deep neural networks, convolutional neural networks, support vector machines, and Bayesian classifiers have high classification accuracy, but they require large-scale CT image datasets with markers of nodular and non-nodular regions to train and are computationally inefficient. As for the rule-based classification method, it primarily establishes a multi-level binary classification system by summarizing the doctor’s diagnosis experience, which has the advantages of high computational efficiency and effective integration of experience. It is well known that for nodule classification and diagnosis, it is critical to detect true nodules as much as possible, thereby ensuring detection sensitivity and accuracy. Thus, the rule-based classification method can be recognized as a useful reference for suspected nodule classification and diagnosis in this work.

### Assisted robot for lung puncture

In the traditional diagnosis and treatment process, if the doctor thinks that the small pulmonary nodules are very likely to be lung carcinomas and meet the indications for surgery, direct surgical resection is usually performed, which is the mature and universal medical treatment. The development of society and advancements in science and technology have increased the demand for precision medicine. Consequently, minimally invasive surgery has made great contributions in the medical field in recent years [2, 25], and percutaneous lung puncture has become the main method of sampling biopsy for suspected pulmonary nodules or tumors. This especially applies in cases wherein the probability of pulmonary nodules as lung cancer is not very high but it is not clear what can confirm the diagnosis not only earlier but at the same time does not cause a relatively large trauma to the patient. Compared with manual puncture [26], robot-assisted percutaneous lung puncture has the advantages of a higher success rate and positioning accuracy and is now the most common technology acceptable to patients.

At home and elsewhere, the study of surgical robots used for percutaneous lung puncture started earlier and developed rapidly, obtaining many good findings, such as a number of products for robot-assisted systems. For example, Ishii et al. [27] developed a remote-controlled 5-degree of freedom (DOF) robot for resolving the radiation issues of CT navigation during the puncture process. Similarly, Hungr et al. [28] designed and validated a percutaneous puncture robot based on CT and MRI navigation and Wang et al. [29] presented an automatic medical tele-robotics system that was used in percutaneous puncture surgery. It should be noted that in this work, we mainly focus on suspected nodule pre-diagnosis and insertion path planning, and will not describe in detail the design and evaluation of surgical robots for minimally invasive percutaneous lungs.

In our previous work, we designed a 6-DOF CT-guided surgical robotic system used for percutaneous lung puncture and manufactured a physical prototype. The relevant technical parameters are as follows: The working ranges of $x,\,y \,and \, z$ axes are 440, 400, and 470 mm, respectively, and the working ranges of $\alpha ,\,\beta \, and \, \gamma$ axes are − 120^○^ to 5^○^, ± 80^○^, and 0^○^–360^*○*^, respectively. The maximum working velocity of linear motion is 0.4 m/s, and the maximum angular velocity of rotational motion is 144^○^/s. Based on the kinematic and inverse dynamic analyses on the designed surgical robot, the Virtual Remote Center of Motion movement of the needle-tip had been achieved by position compensation. For motion control accuracy, it has shown that the position errors of $x,\,y \,and \, z$ axes are 0.4, 0.2, and 0.1 mm, respectively; the posture errors of $\alpha ,\,\beta\, and \, \gamma$ axes are all within 0.2^○^. Moreover, the repositioning accuracy of the robot end is within ± 0.03 mm. Thus, the designed surgical robot satisfied the requirements for practical use.

During actual clinical puncture surgery, in order to make the needle tip successfully reach the lesion point, avoid obstacles such as blood vessels, key tissues, or organs, and reduce the positioning error, insertion path planning is very significant [2, 30, 31]. Unreasonable path planning can reduce puncture accuracy, not only leading to additional tissue damage, but also causing misdiagnosis or unsatisfactory treatment effects [32]. At present, rigid or semi-rigid needles are used in most cases [33, 34] as these have a relatively high hardness, which can reduce the needle deflection caused by straight-line insertion. There have been many studies related to the path planning of flexible needles used in special applications.

From the perspective of algorithms, current insertion path planning methods can be roughly divided into three groups: numerical, search, and inverse solution methods; among these, the numerical methods include probability and objective function methods [35–38].For instance, Lee et al. [36] adopted the path of probability algorithm to develop a systematic planning method for the automated operation of a designed tube-wire flexible needle enabling the needle to easily reach the lesion point while efficiently avoiding obstacles. Fauser et al. [37] presented two suitable RRT-connect algorithms: one based on Bezier–Splines and the other on circular arcs and 3D Dubins Paths, to solve nonlinear path planning for temporal bone surgery with limited space and time constraints. The inverse solution method usually uses the idea of inverse kinematics to calculate the effective trajectory from the entry point to the lesion point; however, the path calculated by this method is not necessarily the optimal one, and there may even be cases where the path cannot be calculated [38]. In this study, a 13G rigid beveled needle manufactured by Precisa Corp., Italia, was used to perform the puncture experiment and validate the effectiveness of the insertion path. Therefore, the objective function method employed in this study can be used to determine and solve the insertion path planning, and this will be described in detail later.

The remainder of this study is structured as follows. First, accurate lung parenchyma segmentation is achieved in chest CT images using digital image processing technologies, such as adaptive gray threshold, connected area labeling, and mathematical morphological boundary repair. Based on lung parenchyma segmentation, the gray judgment criterion is used to extract the suspected nodule areas and analyze their features, including size, shape, and gray value. Next, a multilevel classification criterion is established based on multi-feature weight assignment to realize the automatic pre-diagnosis of nodules. Then, based on a series of CT images stored in DICOM format, we perform the 3-D reconstruction of lung regions using voxelization, and the insertion path planning for the suspected malignant nodule and the appropriate insertion position of the needle on the surface of the thorax are obtained. Finally, some conclusions are drawn that assist doctors diagnose and provide a reference for further study of interaction mechanics between the needle and tissue.

## Materials and methods

In this section, a detailed image-processing method for lung parenchyma segmentation is introduced, including image preprocessing, to improve the quality of the original chest CT images. Subsequently, a multi-feature weight assignment was adopted to establish a multilevel classification criterion to perform the classification and diagnosis of suspected nodules. Additionally, combined with a 3-D model of the reconstructed lung regions, the insertion path planning for the suspected malignant nodule is implemented based on the rule of the shortest path. Of note, we selected publicly available CT datasets from Lung Image Database Consortium and Image Database Resource Initiative (LIDC-IDRI, the most commonly used dataset for early diagnosis of lung cancer) [1, 23, 39, 40] for this study.

### Lung parenchyma segmentation

Figure 1d, e show representative chest CT images. The lung parenchyma represents a darker area in the middle of the original chest image, as shown in Fig. 1d. In fact, lung parenchyma segmentation is the procedure used to extract the left/right lung parenchyma from the background of the original chest image. Figure 1e shows the gray histogram of the chest CT image. It can be seen that the gray-scale distribution appears to be obvious peak-valley-like character. The first peak refers to the external visual field, the second corresponds to the lung parenchyma and air, and the third represents the other lung tissues.

Figure 1f shows a workflow diagram of lung parenchyma segmentation. In addition to the explanatory information, interference factors such as noise exist for the inputted original chest CT images. Therefore, before the initial segmentation, pre-processing, including cropping, enhancing, and denoising the original image, is required. Histogram equalization was used to improve image contrast, and a Gaussian filter was employed to eliminate noise and make the filtered image as close as possible to the original [16, 41, 42]. As a linear smoothing filter, the Gaussian filter is highly effective for suppressing noise that conforms to a normal distribution. The standard deviation of the Gaussian function $\sigma$, determines the width of the filter, and in this study, 6σ was chosen as the total width.

For the initial segmentation of the lung parenchyma, the aim was to classify pixel points whose gray value is smaller than the threshold into lung parenchyma, and the pixel points whose gray value is larger than the threshold into irrelevant areas. Therefore, the effect of the thresholding method depends on the threshold selection. Furthermore, to eliminate the influence of the outside visual field in the CT image on the segmentation, the initial segmentation in this study was performed using the adaptive gray thresholding method, as shown in Fig. 1g. First, the given threshold,${T}_{0}$, is shown in Fig. 1e. The threshold ${T}_{0}$ is determined by the maximum and minimum gray values of the initial image, and the stopping criteria value is 10^–3^. The appropriate threshold is then automatically determined by continuous iteration until the error requirement is met. Finally, the initial segmentation was performed using the final threshold, and the result was the output.

**Fig. 2:**
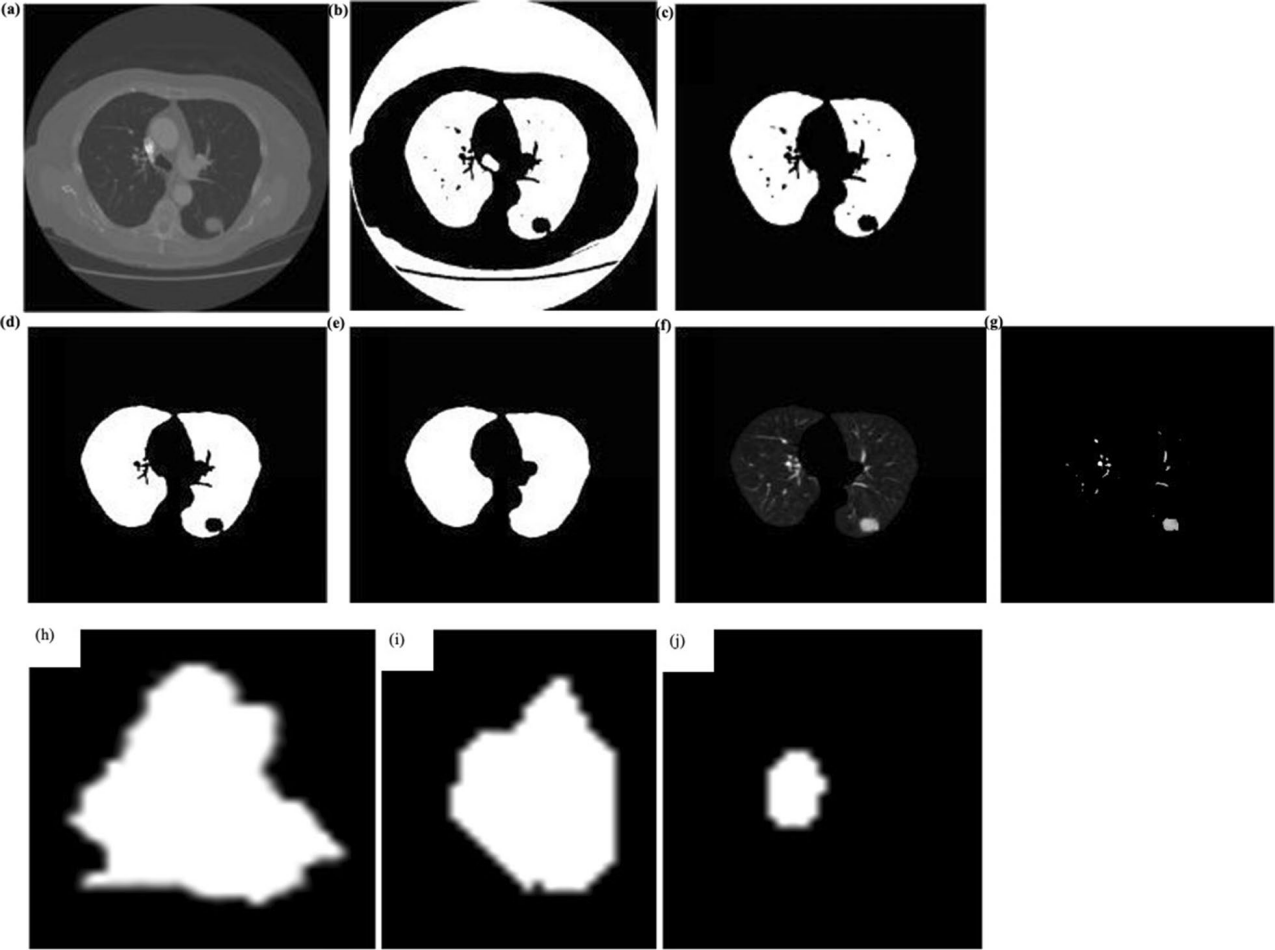
Classification and diagnosis. Results of preliminary segmentation, **a** CT gray image after Gauss filtering. **b** Initial segmentation of lung parenchyma. **c** Left/right lung extracted from initial segmentation of lung parenchyma Lung parenchyma segmentation based on the mathematical morphology, **d** holes filling, **e** boundary repairing, **f** final result of lung parenchyma, **g** possible pulmonary nodules located in segmentation region. **h**–**j** Sketch of some pulmonary nodules

As can be seen from Fig. 2a, b, the output is a binary image. The white area in the middle represents the lung parenchyma. The thresholding method divides parameters such as the air, trachea, into the lung parenchyma, causing interference to further extract the left/right lung. However, there were significant differences in the areas of air, trachea, and lung parenchyma. Generally, the area of the air region is larger than that of the lung parenchyma, and that of the lung parenchyma is much larger than that of the interference region such as the trachea. Thus, based on the area of the connected region, we established a screening criterion to extract the left/right lung. In this study, the 4-neighborhood connected method [43], corresponding to the bwlabel function in MATLAB, was used to mark the connected domain of the binary image (Fig. 2b) and record its area to obtain a set representing the connected domain $\mathbf{s}$. The area set $\mathbf{s}$ is sorted in descending order to obtain the set $\mathbf{S}$, and then the following screening criteria was established based on the set $\mathbf{S}$.

The maximum value of $\mathbf{S}$, the first element of ${S}_{1}$, represents the air region. The second element ${S}_{2}$ and third element ${S}_{3}$ of $\mathbf{S}$ represent the left and right lungs, respectively. The elements after ${S}_{3}$ denote the interference regions, such as the trachea, where the area is relatively small. Therefore, the screening criteria ${S}_{3}$ ≤ s ≤ ${S}_{2}$, is defined to extract the left/right lung. However, considering extreme cases, such as the left and right lung connected as a whole, or only the left lung or the right lung in the field of vision, the screening criteria need to be revised. The scale factor $\uplambda$, is employed to solve this problem. Note that the value of $\uplambda$ is related to the CT image, and in this study, $\uplambda \hspace{0.17em}$= 50 could meet the requirement. When ${S}_{2}/{S}_{3}$ > $\uplambda$, the left and right lungs are connected as a whole or when only one lung is considered the screening criteria is rewritten as s = ${S}_{2}$. Additionally, ${S}_{3}\hspace{0.17em}$≤ s ≤ ${S}_{2}$ is universally applicable. Figure 2c shows the extracted result for the left/right lung from the binary image.

Because the lung contains complex tissues such as blood vessels, more holes appear inside the extracted lung parenchyma, as depicted in Fig. 2c. Moreover, there may be a phenomenon wherein the blood vessels, pulmonary nodules, and contour of the lung parenchyma are adhered to the lung, so that the extracted lung parenchyma is defective leading to missed detection with a high probability in later work. Thus, it is necessary to perform internal hole filling and boundary repair during the initial segmentation of the lung parenchyma. In our work, we complete the above two steps based on mathematical morphology, and then multiply the binary image of repaired lung parenchyma segmentation and the pre-processing CT gray image to obtain a complete segmentation.

The specific process was as follows: First, we adapted the morphological reconstruction method to realize hole filling. Specifically, the pixel value of the hole area is changed up to its boundary by calling the imfill function in MATLAB software, as shown in Fig. 2d, and the mathematical morphology is used to perform the boundary repair. In other words, boundary repair can be achieved by various operations, such as corrosion, expansion, opening, and closing (morphology) operations [44, 45]. The morphological operators are named erosion and dilation, which can be used one after the other, namely opening is erosion + dilation; and closing is dilation + erosion. Considering that in the actual repair process, there is a case where adhesion is likely to occur when the distance between the left and right lungs is relatively close. Therefore, we individually repaired and combined the separated left and right lungs. The structural elements of the opening operation and corrosion operation are first constructed, and then for the left/right lung, the boundary is repaired by the opening operation, and the smoothing of the boundary is achieved by the corrosion operation. Figure 2e shows the mask of the lung parenchyma obtained after combining the results for the left and right lungs. Figure 2f illustrates the final result of the lung parenchyma, which was obtained by multiplying Fig. 2a, e. It can be seen that the gray-scale of the lung parenchyma is significantly different. For most regions of the whole image, the gray scale is lower, but the gray scale of pulmonary nodules or blood vessels is higher and brighter.

1 $$Dice\left( {S1,S2} \right) = 2 \times \frac{{\left| {S1 \cap S2} \right|}}{{\left| {S1} \right| + \left| {S2} \right|}}$$

where $S_{1}$ is the final result of lung parenchyma segmentation using the method established in this study and $S_{2}$ is the manual segmentation of the lung parenchyma.

In addition to the subjective observation with the naked eye, manual segmentation of original CT images was used as a reference, and an assessment of lung parenchyma segmentation was conducted by calculating the dice similarity coefficient, defined in Eq. (1). The sample is selected as 400 random images, and the accuracy of the calculated lung parenchyma segmentation was 97.4%. Next, compared with the other methods for lung parenchyma segmentation, such as the adaptive concave hulls with active contours and the graph cu, it can be noted that the accuracy in this study is lower than 98.07% as reported in one study but greater than 95.9% documented in another report [46, 47]. Our results indicate that the performance of the established method for lung parenchyma segmentation is acceptable, which could assist in building the region of interest used to search for when identifying the nodules, and be convenient for generating 3D visualizations for path planning.

### Classification and diagnosis

Based on lung parenchyma segmentation, we then classified and diagnosed pulmonary nodules to segment the suspected nodule region. The adaptive grey thresholding method was used to make an initial selection of the suspected nodule region, as shown in Fig. 2f. Then, a formula for gray judgment was employed and added to each initial selected nodule region to optimize the boundaries of the suspected nodule region. Based on the idea of regional growth, the formula for gray judgment can be defined as Eqs. (2), as shown below,

2 $$g\left( {i,j} \right) = \left\{ {\begin{array}{*{20}l} {g\left( {i,j} \right)} \hfill & { if\quad g\left( {i,j} \right) \ge \left( {1 - \tau } \right)*\frac{{\left( {g_{max} + g_{min} } \right)}}{2}} \hfill \\ 0 \hfill & {else.} \hfill \\ \end{array} , } \right.$$

where $g\left( {i,j} \right)$ represents the gray value at pixel points of $\left( {i,j} \right)$, $g_{max}$ is the maximum gray value in a single suspected nodule region, $g_{min}$ is the minimum gray value in a single suspected nodule region, and $\tau$ is the adjustment parameter which varies from 0 to 1. The $\tau$ is a variable value, which depends on image characteristics. Usually, we first set it to 0.5, and then try other values.

Figure 2g shows the segmentation results for the possible pulmonary nodules. It can be seen that the regions of possible pulmonary nodules contain components such as nodules, vessels; therefore, further screening is needed to combine the clinical features of pulmonary nodules.

Clinical experience has shown that malignant pulmonary nodules are often characterized by irregular edges accompanied by protrusions and burrs, uneven distribution of internal grayscale, large volume, and diameter generally exceeding 3 mm; however, benign pulmonary nodules present smooth edges with no obvious protrusions and burrs. In view of the above, a multilevel classification criterion has been established based on the shape and grayscale features of pulmonary nodules [48, 49].

The shape features are the most intuitive features of pulmonary nodules. The size of the pulmonary nodule and the marginal burr are the main features that characterize benign and malignant nodules, and quasi-roundness can be used to differentiate round-like nodules and strip vessels owing to their difference in circular proximity. In this study, four shape features of area $\left( {S_{R} } \right)$, quasi-roundness $\left( {C_{R} } \right)$, marginal burr rate $\left( {MBR} \right)$, and marginal burr magnitude $\left( {MBM} \right)$, were selected to describe the size, shape, and edge information of nodule region $\left( R \right)$.

The nodule region $\left( R \right)$ is irregular in shape, and calculation of its real area is difficult. Thus, the total number of pixels contained in the region is used to characterize the area $S_{R}$, which can be expressed as $S_{R}$ = $Count\left( R \right)$. The larger the area, the higher the probability that the region is a malignant nodule. The quasi-roundness $\left( {C_{R} } \right)$, indicates the proximity of the region to the circle, which can be expressed as Eq. (3), as shown below,

3 $$C_{R} = \frac{{4\pi S_{R} }}{{(P_{R} )^{2} }}$$

where $P_{R}$ is the perimeter of region $\left( R \right)$, that is, the length of the region boundary.

The marginal burr rate, $MBR$, is the ratio of the number of burrs on the boundary ($n_{b}$) to the total number of boundary points ($n$), which is defined as Eq. (4), as shown below,

4 $$MBR = \frac{{n_{b} }}{n}$$

The marginal burr magnitude, *MBM*, represents the mean of all changes in the radial distance at each burr point relative to the average radial distance of the boundary, which can be written as Eq. (5), as shown below,

5 $$MBM = \frac{{\mathop \sum \nolimits_{i = 1}^{{n_{b} }} [RD\left( i \right) - RD\left( {ave} \right)]^{ } }}{{n_{b} }}$$

with Eqs. (6), as shown below,

6 $$\left\{ {\begin{array}{*{20}l} {\overline{RD} \left( i \right) = \sqrt {(\left( {x\left( i \right) - x_{R} } \right)^{2} + \left( {y\left( i \right) - y_{R} } \right)^{2} } } \hfill \\ {RD\left( i \right) = \frac{{\overline{RD} \left( i \right)}}{{\mathop {\max }\limits_{ } \left\{ {\overline{RD} \left( i \right),i = 1, \ldots ,n} \right\}}}} \hfill \\ {RD\left( {ave} \right) = mean\left( {RD\left( i \right)} \right),\left( {i = 1, \ldots ,n} \right)} \hfill \\ \end{array} } \right.$$

where $\left( {x_{R} ,\left. {y_{R} } \right)} \right.$ is the centroid coordinates of region $R$, $RD\left( i \right)$ is the normalized distance from the boundary point of region $\left( R \right)$ to the center of mass of region $\left( R \right)$, and $RD\left( {ave} \right)$ is the average radial distance of all boundary points.

In general, for nodule region $\left( R \right)$, the larger *MBR*, the greater the probability that it is a true positive nodule, and the larger the *MBM*, the greater the probability that it is a malignant nodule.

Gray-scale features are simple to calculate and are not sensitive to image translation, rotation, and scaling. To describe the distribution characteristics of grayscale in region $\left( R \right)$, three gray-scale features, gray mean $\left( {GM} \right)$, gray variance $\left( {GV} \right)$, and gray entropy $\left( {GE} \right)$, are selected.

For each pixel of region $R$, its grey value can be directly read and obtained. Therefore, the gray mean $GM$ can be expressed as Eq. (7), as shown below,

7 $$GM = \frac{{\mathop \sum \nolimits_{{\left( {i,j} \right) \in R}} g\left( {i,j} \right)}}{{S_{R} }}$$

The gray variance, $GV$, indicating the deviation amplitude between the gray-scale distribution and gray mean in region $\left( R \right)$, can be written as Eqs. (8), as shown below,

8 $$GV = \frac{{\mathop \sum \nolimits_{{\left( {i,j} \right) \in R}} \left[ {g\left( {i,j} \right) - GM} \right]^{2} }}{{S_{R} }}$$

The gray entropy, $GE$, which indicates the amount of grayscale information of the image, can be expressed as Eq. (9), as shown below,

9 $$GE = - \mathop \sum \limits_{k = 0}^{255} \frac{A\left( k \right)}{{S_{R} }}\log \left( {\frac{A\left( k \right)}{{S_{R} }}} \right)$$

where $A\left( k \right)$ represents the number of pixels in region $\left( R \right)$ whose gray value is $k$.

Based on the shape and grayscale features mentioned above, we performed feature extraction of pulmonary nodules confirmed by clinical practice. Table 1 shows the feature data of some pulmonary nodules, as shown in Fig. 2h–j.

**Table 1:** Extracted features of some pulmonary nodules

Sketch of pulmonary nodules	Shape features				Gray-scale features		
	$\left( {S_{R} } \right)$	$\left( {C_{R} } \right)$	*MBR*	*MBM*	$\left( {GM} \right)$	$\left( {GV} \right)$	$\left( {GE} \right)$
(a)	846.5	0.54	0.28	0.13	100.66	126.41	4.42
(b)	271.5	0.7653	0.19	0.10	139.02	119.49	4.84
(c)	39.0	0.858	0.41	0.13	95.24	77.74	4.50

Based on the extracted features of doctor-confirmed pulmonary nodules (Table 1), a multilevel classification criterion was established to perform the classification and recognition of suspected pulmonary nodules, thereby assisting the doctor in completing the diagnosis. The classification criteria results are collected from the selected datasets, i.e., shape and grayscale feature data of pulmonary nodules marked by doctors. Specifically, the classification criteria included the following:

1. As described previously [1], the identified lesions are usually marked by radiologists as non-nodules, < 3 mm nodules, and ≤ 3 mm nodules. Since nodules < 3 mm are mostly benign and are difficult to diagnose automatically, the subsequent sample statistics are ≤ 3 mm nodules. The threshold of the area $\left( {S_{R} } \right)$, $T_{S}$, was defined to distinguish nodules and eliminate interference factors, such as nodules < 3 mm.
2. The image of the nodule in the CT cross-section was round. The threshold of quasi-roundness $\left( {C_{R} } \right)$, $T_{C}$, was employed to distinguish between the nodules and elongated vascular tissues.
3. The most significant interference factor for nodule classification is the larger vascular tissue perpendicular to the CT cross-section, and its image also appears to be round-like. However, compared with the nodules, the blood vessels of this type have larger grayscales and an uneven gray distribution, and the boundaries are smoother. Hence, the threshold of the gray mean $\left( {GM} \right)$, $T_{GM}$, the threshold of the gray entropy $GE$, $T_{GE}$, and the threshold of the marginal burr rate $\left( {MBR} \right)$, $T_{MBR}$, can be used to distinguish between the nodules and larger vascular tissue.
4. The large nodules with diameters > 10 mm, corresponding to the area of approximately 300, are highlighted for medical reference.
5. The key procedure for classification is selection with $T_{S}$, $T_{C}$, $T_{GM}$, $T_{GE}$ and $T_{MBR}$. In our previous work, the above multi-level classification parameters were normalized based on the statistical theory, and a better combination was obtained based on the 3σ rule; however, for the first two types of pulmonary nodules (as seen in Fig. 1a–c), the classification quality was not good. Hence, we used the weighted average method for the above parameters to complete the multi-feature weight assignment, that is, $T_{i} = \xi_{i} *T_{i} \left( {i = S, C, GM, GE, MBR} \right)$, where $\sum \xi_{i} = 1$, and the neural network algorithm was used to search for global optimal solutions.

Based on the above criteria, the final optimization results are $T_{S}$ = 30, $T_{C}$ = 0.6, $T_{GM}$ = 196, $T_{GE}$ = 3.5, and $T_{MBR}$ = 0.16. In addition, a confusion matrix was used to describe the performance of the suspected nodule pre-diagnosis established in this study.

It was assumed that the nodule was defined as positive and the non-nodule was considered as negative. $$TP$$ indicates that the diagnosis is positive and itself is positive. False positive ($$FP)$$ indicates that the diagnosis is positive and itself is not positive. $$TN$$ indicates that the diagnosis is negative, and itself is negative; in this case, $$TN$$ = 0. False negative ($$FN)$$ indicates that the diagnosis is negative and itself is not negative. Therefore, four evaluation metrics–sensitivity $$(F_{se}$$), specificity $$(F_{sp}$$), accuracy $$(F_{a}$$), and computational efficiency $$(F_{ce}$$)–were employed and used, as shown in Eq. (10).

10 $$F_{se} = \frac{TP}{{TP + FN}},F_{sp} = \frac{TN}{{FP + TN}}, F_{a} = \frac{TP}{{TP + TN + FP + FN}},F_{ce} = t.$$

where $t$ is the time required to process a CT image. Note that $F_{sp}$ = 0 because $TN$ = 0.

### Insertion path planning

The results of the automatic classification and diagnosis of suspected pulmonary nodules in the previous section can not only be used as a pre-diagnosis before the final diagnosis by doctors, but also further assist them in determining the location of the suspected malignant nodules. For cases in which benign and malignant tumors cannot be diagnosed, biopsy puncture is required. As a result, finding an optimized feasible insertion path is another important aspect of this study.

3-D reconstruction technology based on 2-D medical images can better express the form, structure, and spatial relationships, providing further image information for biological medical studies [50, 51]. Obviously, the intuitive 3-D structure and its adjacent relationship are of great benefit to the insertion path planning of biopsy puncture. It is not difficult to imagine that designing the insertion path based on the 3-D model is very likely to be significantly similar to clinical practice, such as whether key tissues are avoided, the feasibility of inserting points, and so on, which does not include the influencing factors such as breathing, heartbeat, in puncture research [2, 34]. Therefore, we should first obtain the 3-D model of the reconstructed lung regions to design and complete reasonably optimized path planning.

The main steps of 3-D reconstruction are as follows: First, a series of 2-D chest original images require preprocessing, such as image enhancement and denoising, which is the same as the previous lung parenchyma segmentation. Second, threshold, morphology, and boundary repair operations are performed on the pre-processed images to extract the lung parenchyma, as described in Sect. "Lung parenchyma segmentation". Based on lung parenchyma segmentation, the point cloud was calculated using voxelization to obtain 3-D volume data. Affine transformation was also used for the volume data to get interpolated 3-D volume data. Third, the MC algorithm (i.e., the marching cubes algorithm [52] was adopted to perform the 3-D reconstruction of the interpolated 3-D volume data. Although the point cloud reconstruction algorithm [53] in authors’ work can make the reconstruction effect better, the common method we adopted has met the clinical requirements, so we did not pursue higher accuracy requirements. After extracting the iso surfaces, a triangular patch with the same threshold is formed, the smoothing process is performed, and the lighting model is used for shading rendering.

**Fig. 3:**
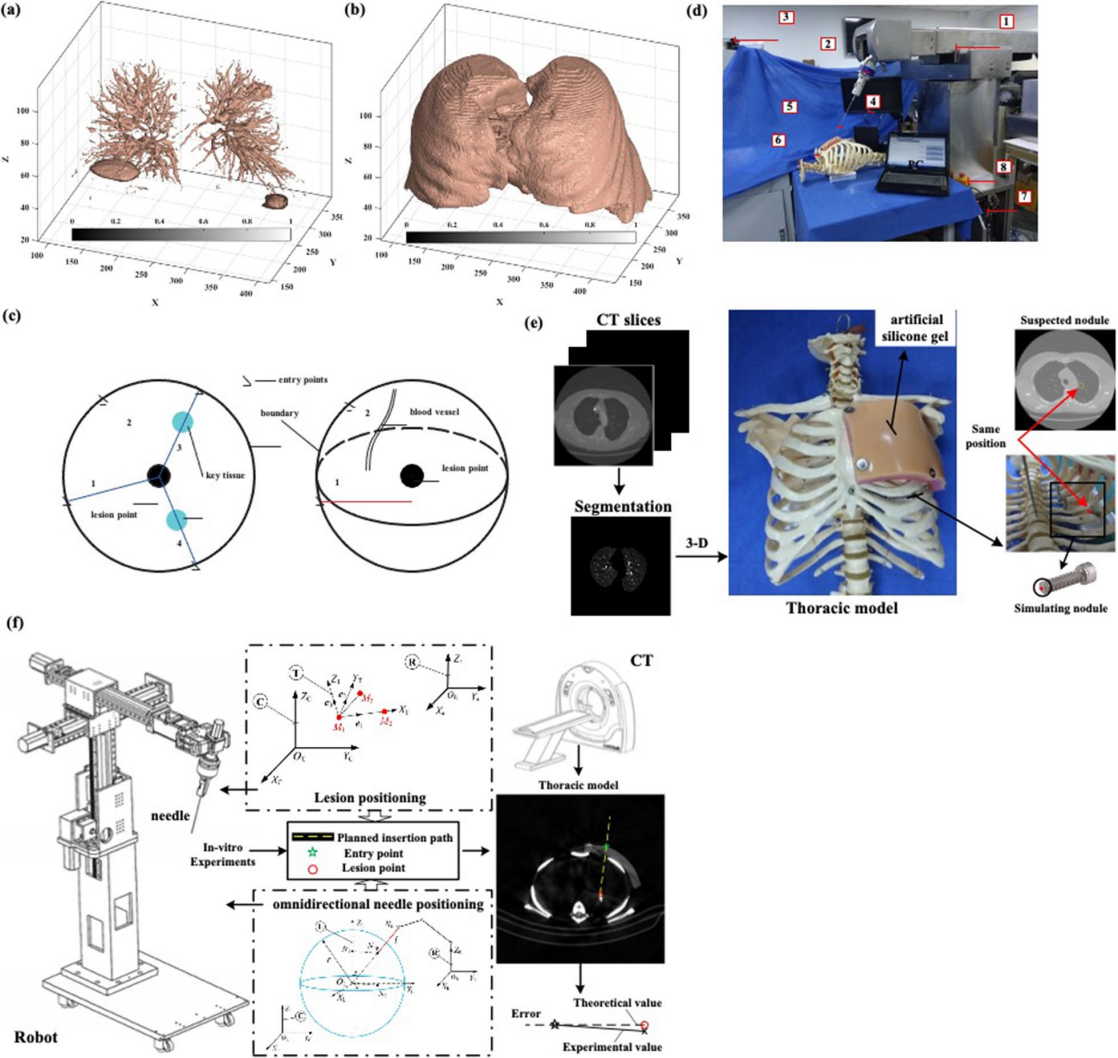
Insertion path planning. 3-D model of reconstructed lung regions: **a** internal tissues, and **b** whole chest model. **c** Illustration of finding the optimize feasible insertion path. **d** Designed surgical robotic system used for minimally invasive percutaneous lung. The numbers represent as below: 1-noumenon structure, 2-force sensor, 3-camera, 4-video surveillance, 5-needle, 6-thoracic model, 7-controller, and 8-emergency stop button. **e** Graphic of simulating nodule used to represent suspected nodule in experiment. **f** Description of in-vitro puncture experiments for verifying path planning

By selecting an appropriate gray threshold, 3-D reconstruction models of different tissues or pulmonary nodules can be achieved. Figure 3a shows the internal tissues, such as the blood vessels, tracheae, and pulmonary nodules. Different selections of the grey threshold can clearly distinguish these internal tissues. The entire chest model referring to the lung regions is depicted in Fig. 3b. The thoracic ribs can be clearly seen, which is useful for inserting a path design for biopsy puncture.

According to clinical experience, the insertion path planning for biopsy puncture needs to follow two principles: one to observe the initial entry point in detail through the 3-D reconstruction model to reasonably avoid key tissues or organs, and the other to shorten the puncture distance as much as possible under the premise of effective obstacle avoidance. Thus, the optimal puncture path should theoretically select the shortest distance from the center of the mass of the nodule to the external boundary surface of the chest model. The shortest distance of the puncture path, $d_{min}$, is expressed as:

11 $$\left\{ {\begin{array}{*{20}l} {d_{min} = min\left\{ {\sqrt {\left( {x_{i} - x_{l} } \right)^{2} + \left( {y_{i} - y_{l} } \right)^{2} + \left( {z_{i} - z_{l} } \right)^{2} } } \right\}} \hfill \\ {S_{c} = \left\{ {\left( {x_{i} ,y_{i} ,z_{i} } \right) \in B_{c} } \right\},\left( {i = 1,2, \ldots ,n_{c} } \right)} \hfill \\ \end{array} } \right.$$

where $\left( {x_{l} ,y_{l} ,z_{l} } \right)$ are the centroid coordinates of the suspected pulmonary nodule; $\left( {x_{i} ,y_{i} ,z_{i} } \right)$ are the coordinates of the boundary point on the external surface of the chest model ($B_{c}$); $S_{c}$ represents an aggregate of all boundary points; and $n_{c}$ is the number of all boundary points.

It can be calculated using Eq. (11), the boundary points corresponding to the shortest distance of the puncture path are the designed insertion points (i.e., the initial entry points), and the lines connecting the insertion points to the center of the suspected pulmonary nodule are the optional insertion paths. In this study, we first found the chest CT slice on which the most suspected malignant nodule was displayed, then established the objective function of the sum of straight-line pixels on this slice to search for the minimum value, and finally determined the feasible solution based on the 3-D reconstruction model, thereby obtaining the optimal insertion path. Figure 3c illustrates the optimized feasible insertion path. Based on the objective function method, the mathematical description for determining the optimal insertion path can be expressed as

12 $$\left\{ {\begin{array}{*{20}l} {g_{min} = min\sum g\left( {x_{i} ,y_{i} ,z_{i} } \right)} \hfill \\ {s.t.\left( {x_{i} ,y_{i} ,z_{i} } \right) \in d_{min} } \hfill \\ \end{array} } \right.$$

where $g\left( {x_{i} ,y_{i} ,z_{i} } \right)$ represents the gray value at pixel points of $\left( {x_{i} ,y_{i} ,z_{i} } \right)$ which belongs to the insertion path (that is, the shortest straight line calculated from Eq. (11)), $g_{min}$ is the minimum gray value of the sum of the pixels.

As shown in Fig. 1d, e, different tissue structures have different grey values. In other words, the sum of the gray values of all the pixels on different insertion paths is different. It can be seen from Fig. 3c that at the CT slice on which the largest size of the suspected malignant nodule is displayed, there could be four shortest paths solved by Eq. (11); however, from Eq. (12), it can be found that the sum of the gray values of Nos. 1 and 2 is smaller than that of Nos. 3 or 4. Subsequently, it is necessary to combine the established 3-D reconstruction model, ensuring that key blood vessels are completely avoided, and the insertion path (No. 1) can be determined as the optimal insertion path. Thus, without considering the relevant influencing factors in the puncture study, Eqs. (11–12) and the 3-D reconstructed model, a better insertion path can be found by avoiding the sternum and/or key tissues from the aspect of path planning. A MATLAB program was developed to solve these equations.

Based on our previous work for minimally invasive percutaneous lung, as shown in Fig. 3d [54], the designed surgical robotic system was used to perform in vitro experiments and to verify the effectiveness of insertion path planning for biopsy puncture. The main technical parameters and the performance of the designed robotic surgical system are introduced in Sect. "Assisted robot for lung puncture". The robot design and prototype manufacturing are not described here, the details are as in the earlier study [54].

It should be noted that in this study, a thoracic model from the 3-D reconstructed model of one patient was custom-made, mainly consisting of a human skeleton model on a 1:1 scale and an artificial silicone gel with three layers used to simulate soft tissue on the surface of the thoracic ribs. After determining the positions of suspected pulmonary nodules from CT slices, the simulating nodule points (the lesion points) were set inside the model using bolts of different lengths, where the coordinates of the end center point of the bolts were the same as the positions of the suspected pulmonary nodules (Fig. 3e). Although our previous work involving in vitro puncture experiments has been published [2, 54], the necessary description is explained below for easy understanding of the readers, and also shown in Fig. 3f.

For robot-assisted puncture, lesion positioning and needle positioning are the basis to ensure the accuracy of the puncture and are the key techniques for insertion operation. As per a report [54], a lesion positioning method based on three noncollinear markers aiming to be realized only by the robot-CT system without using an external positioning system was established. Using this lesion positioning method, the needle tip can easily reach the initial entry point, that is, the entry point of the planned insertion path. In a previous study [54], an omnidirectional needle positioning method was developed and realized using the Virtual Remote Center of Motion. Using this needle-positioning method, the needle axis can always be along the direction of the planned insertion path. It should be noted that the shortest distance and smallest gray-value sum for the planned insertion path can, theoretically (Eqs. (11–12) and visually (3-D model) avoid obstacles, such as the key blood vessels. In an actual insertion operation, the azimuth angle of the puncture needle in the omnidirectional needle positioning method can easily be adjusted to avoid unexpected obstacles in the planned insertion path.

It is well known that the coordinates of the suspected pulmonary nodule (the lesion point) in the CT coordinate system can be directly output from the above nodule recognition result. The specific verification steps for the insertion path are as follows: First, by using the lesion positioning method, the positioning of the lesion point in the robot coordinate system can be obtained, and the initial entry point and insertion path can be mapped to the robot coordinate system. Subsequently, after using the designed surgical robotic system to finish the in vitro puncture experiments, the effectiveness of the insertion path can be verified by comparing the error between the theoretical and actual experimental positions of the lesion point.

## Results and Discussion

The publicly available database of chest CT images from LIDC-IDRI was used to verify the validity of the established method for pulmonary nodule pre-diagnosis. Moreover, a CT-guided surgical robotic system used for minimally invasive percutaneous lung and a custom thoracic model were designed and made to perform in vitro puncture experiments to validate the effectiveness of the insertion path planning.

### Pulmonary nodule detection

**Fig. 4:**
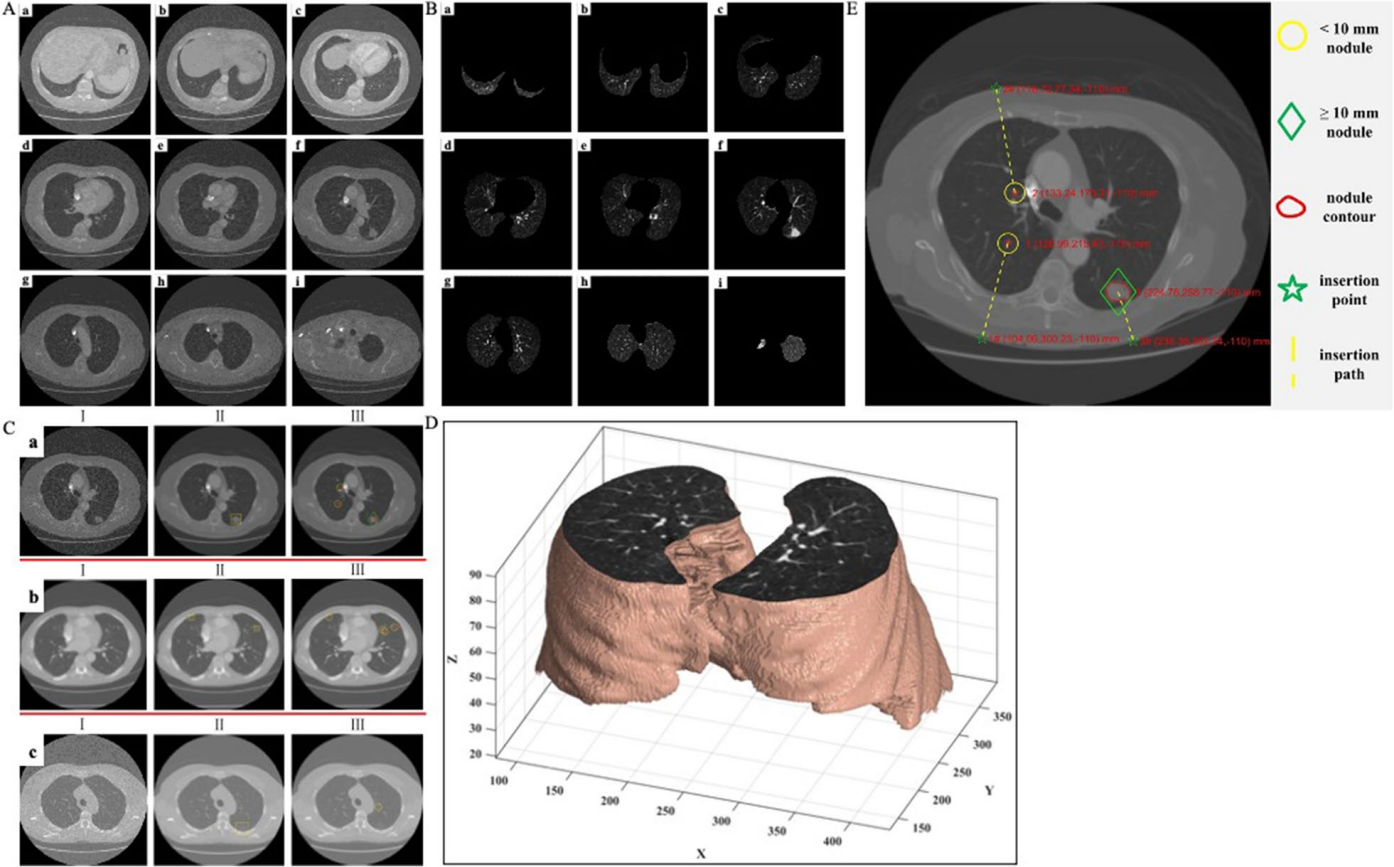
Pulmonary nodule detection. **A** CT gray images at different slices for the same patient. **B** Results of lung parenchyma segmentation corresponding to gray images in Fig. 4A. **C** Automatic recognition experiments for pulmonary nodules, where I to III represent original images, doctor-marked nodules, and diagnostic results in this paper, respectively. **D** A cross-section of the reconstructed 3-D model. **E** Path planning of nodules used for biopsy puncture.

LIDC-IDRI contains a series of original CT images of over 1000 patients, and the median slice thickness of the images with an interval of [0.6, 5.0 mm] is approximately 2 mm. Taking one of the patients as an example, nine original CT images at equal intervals in the lung region, as shown in Fig. 4A, were selected to extract the lung parenchyma segmentation. Then, the CT images were processed using the established image processing method for lung parenchyma segmentation as discussed in Sect. "Lung parenchyma segmentation". The obtained results are shown in Fig. 4B.

As shown in Fig. 4B, all CT images successfully segmented the lung parenchyma with clear edges, which removed the background of the thoracic contour in the original images. As the experience we met in the process of work, the repair effect in the case of contour adhesion among blood vessels, pulmonary nodules, and lung parenchyma was better, and the loss rate of gray information in the extracted lung parenchyma was lower. In other words, the lung parenchyma in chest CT images can be accurately identified using the established method for lung parenchyma segmentation, which is useful for ensuring the effects of later pulmonary nodule detection.

Based on lung parenchyma segmentation, the classification and diagnosis of pulmonary nodules were performed on CT images of a total of 900 patients. The four-evaluation metrics in Eq. (10), sensitivity (*F*_se_), specificity (*F*_sp_), accuracy (*F*_a_), and computational efficiency (*F*_ce_) were obtained after completing all studies on pulmonary nodule detection. Figure 4C shows some representative automatic recognition experiments for pulmonary nodules.

After the comprehensive analysis, we draw the following conclusions:

1. The time for processing a single CT image is about 5 s, i.e., the computational efficiency, *F*_ce_, is relatively high.
2. Unlike the definition of negative for benign and malignant detection of nodules, in this study, the non-nodule is defined as negative, TN = 0, so the specificity, *F*_sp_, is 0.
3. The average sensitivity, *F*_se_, was 88.2%, indicating that only 11.8% of all the annotated nodules remained unrecognized, and a few errors occurred in unclear CT images.
4. The average accuracy, *F*_a_, was 82.1%, indicating that although the established suspected nodule pre-diagnosis method was able to recognize the vast majority of all the nodules in 900 patients, the number of false positives (*FP*) was slightly higher.

As mentioned in the Introduction section, in recent years, most of the published literature on the detection of pulmonary nodules has been based on machine learning for benign and malignant detection of pulmonary nodules, and there is few research on recognizing nodules. Here, compared with the other methods for pulmonary nodule recognition, such as the multi-view convolutional networks (ConvNets) [39] and the front-end detector/segmentor [55], it was seen that the sensitivity (*F*_se_) of nodule recognition in our work is larger than 81.3% reported [39], but the improvement of the candidate detection algorithm based on multi-view ConvNets as proposed by Setio et al. could reach a higher detection sensitivity (approximately 90.1%), which would be suited to be used for FP reduction and might give us a reference to reduce FP in our follow-up work. Additionally, the sensitivity (*F*_se_) in our work was > 82.66% documented earlier [55]. Although the accuracy (*F*_a_) was lower than 92.8% reported [55], the LIDC data used in that study just comprised of 84 CT scans that contained only 143 nodules, and for different selected features, the accuracy changed from 92.8% to other values [80.4% of the nodules (115/143)] using 40 features. Obviously, the comparison of the magnitude of accuracy, or for that matter, the other evaluation metrics, is only a qualitative analysis, and there are many differences pertaining to the conditions that prevailed for varied research focuses such as only for recognizing solitary pulmonary nodules. Similarly, even in the same datasets, sample selection and the amount also contribute to problem of randomness, and cannot be completely consistent.

In summary, the accuracy shown in the results is not only related to the recognition of small pulmonary nodules, but also to the misidentification of blood vessels, in which the difference in features between the nodules and the vessels perpendicular to the CT cross-section is not fully excavated. However, combined with the feedback from senior experts, it can be concluded that although the average sensitivity and accuracy are 88.2% and 82.1%, respectively, the results can basically meet clinical requirements. Subsequently, in further 587 patients, we plan to apply the generative adversarial network algorithm to achieve medical image data augmentation [56, 57], and thus to pursue better detection of pulmonary nodules.

### Path planning

Figure 4D shows the cross section of the reconstructed 3-D model. Based on the 3-D reconstructed model, we can easily and quickly check the segmentation of the lung parenchyma in any cross-section and the spatial position of each structure. This also allows for the effective screening of optional insertion paths.

Figure 4E depicts an example of path planning of nodules used for biopsy puncture. The green five-pointed star indicates the insertion point corresponding to each nodule, and the yellow dotted line represents the optimal insertion path. The centroid coordinates of each nodule in the CT coordinate system are given along with those of the insertion point. Next, the lesion positioning error was used to evaluate the effectiveness of the designed insertion path. Experimental studies have shown that the positioning error of each lesion are 2.015, 2.421, and 2.145 mm, respectively, and are within 3 mm. This also reveals the validity of the path planning, which can provide reference for clinical biopsy puncture [58].

This study demonstrates that the established automatic pre-diagnosis method, including lung parenchyma segmentation, classification, and pre-diagnosis, is effective for the recognition or detection of suspected pulmonary nodules, regardless of the benign and malignant nodules, and that the use of a CT-guided surgical robotic system and a custom thoracic model is feasible for the validation experiment of insertion path planning. However, there are still some limitations as discussed below:

1. While the results were observed from the CT images of 900 patients, the performance of the automatic pre-diagnosis method in this study has scope for further improvement. The evaluation metrics (i.e., *F*_*se*_*, F*_*a*_) imply that it is able to recognize the vast majority of all annotated nodules, but the number of FPs should be reduced based on the latest advances in AI, such as deep learning, and warrants continued future research.
2. The experiment for verifying insertion path planning in this work is an in vitro study, performed in a custom thoracic model and an artificial silicone gel with three layers, with no in vivo or ex vivo effects in humans or animals. It is well known that all the results observed from in vivo human experiments are the most convincing, but they are difficulties in implementing them both practically and ethically. Therefore, it would be interesting to explore the extent to which ex vivo animal experiments can be used and the artificial biological tissue that can be prepared for follow-up work.

For the early diagnosis and screening of lung carcinoma, accurate detection of pulmonary nodules is currently the most effective approach for preventing and providing reliable clinical treatment schema. In this study**,** we explored the automatic pre-diagnosis of suspected nodules and insertion path planning of suspected malignant nodules for biopsy puncture. The overall process includes lung parenchyma segmentation, classification, pre-diagnosis, 3-D reconstruction, and path planning. The extraction of lung parenchyma from chest CT images is achieved using digital image processing technologies, such as adaptive gray threshold, connected area labeling, and mathematical morphological boundary repair. A multilevel classification criterion is established based on the shape and grayscale features of pulmonary nodules, and the multi-feature weight assignment is used to determine the thresholds of selected parameters, where the global optimal solutions are searched using a neural network algorithm. Based on these, the classification and pre-diagnosis of suspected nodules can be obtained. Although doctors still need to be involved in judging benign and malignant nodules, the automatic pre-diagnosis process in this work has greatly reduced the doctor’s diagnostic burden. Finally, 3-D reconstruction of lung regions is performed using voxelization, and on this basis, the optimized feasible insertion path with an insertion point can be found by avoiding sternums and/or key tissues in path planning. Additionally, in vitro puncture experiments were conducted to validate the effectiveness of planned insertion path. It can be concluded that the lung parenchyma segmentation in this study is acceptable, which is helpful in building the region of interest used to identify nodules. The average sensitivity and accuracy are 88.2% and 82.1%, respectively. The established suspected nodule pre-diagnosis method can recognize vast majority of all nodules in 900 patients, but the number of FPs was slightly higher. Nevertheless, combined with feedback from senior experts, the results can meet clinical requirements. In future work, the generative adversarial network is planned to adapt to pursue better accuracy with fewer FPs. Moreover, as experiments showed the position errors of lesion points after insertion to be within 3 mm, revealing the validity of path planning can provide a reference for clinical biopsy puncture.

## Data Availability

The datasets analysed during the current study available from the corresponding author on reasonable request.

## References

[1] Zhang J, Xia Y, Cui H, Zhang Y (2018). Pulmonary nodule detection in medical images: a survey. Biomed Signal Process.

[2] Chen GB, Fu Z, Zhang TF, Shen Y, Wang Y, Shi W, Fei J. Robot-assisted puncture positioning methods under CT navigation. J Xi’an JiaoTong Univ. 2019;53:85–92, 99.

[3] Bray F, Ferlay J, Soerjomataram I, Siegel RL, Torre LA, Jemal A (2018). Global cancer statistics 2018: GLOBOCAN estimates of incidence and mortality worldwide for 36 cancers in 185 countries. CA Cancer J Clin.

[4] Baldwin DR (2015). Prediction of risk of lung cancer in populations and in pulmonary nodules: significant progress to drive changes in paradigms. Lung Cancer.

[5] Bo L, Li C, Pan L, Wang H, Li S, Li Q (2019). Diagnosing a solitary pulmonary nodule using multiple bronchoscopic guided technologies: a prospective randomized study. Lung Cancer.

[6] Zhang R, Tian P, Chen B, Zhou Y, Li W (2020). Predicting lung cancer risk of incidental solid and subsolid pulmonary nodules in different sizes. Cancer Manag Res.

[7] Kadir T, Gleeson F (2018). Lung cancer prediction using machine learning and advanced imaging techniques. Transl Lung Cancer Res.

[8] Krishnamurthy S, Narasimhan G, Rengasamy U (2015). Three-dimensional lung nodule segmentation and shape variance analysis to detect lung cancer with reduced false positives. Proc Inst Mech Eng Part H J Eng Med.

[9] Tao G, Jingying Y, Tan G, Xiaotao D, Min C, China D of RBH National Center of Gerontology, Beijing (2018). A novel CT-guided technique using medical adhesive for localization of small pulmonary ground-glass nodules and mixed ground-glass nodules (≤20 mm) before video-assisted thoracoscopic surgery. Diagn Interv Radiol.

[10] Reilly RM. Medical Imaging for Health Professionals. 2019:1–9.

[11] Ohno Y, Kauczor H, Hatabu H, Seo JB, Beek EJR, (IWPFI) for the IW for PFI (2018). MRI for solitary pulmonary nodule and mass assessment: current state of the art. J Magn Reson Imaging.

[12] Bamji-Stocke S, van Berkel V, Miller DM, Frieboes HB (2018). A review of metabolism-associated biomarkers in lung cancer diagnosis and treatment. Metabolomics.

[13] Ambrosini V, Nicolini S, Caroli P, Nanni C, Massaro A, Marzola MC (2012). PET/CT imaging in different types of lung cancer: an overview. Eur J Radiol.

[14] John J, Mini MG (2016). Multilevel thresholding based segmentation and feature extraction for pulmonary nodule detection. Proc Tech.

[15] Khan ZF (2019). Automated segmentation of lung parenchyma using colour based fuzzy C-means clustering. J Electr Eng Technol.

[16] Javaid M, Javid M, Rehman MZU, Shah SIA (2016). A novel approach to CAD system for the detection of lung nodules in CT images. Comput Methods Programs Biomed.

[17] Sridhar B, Reddy KVVS, Prasad AM (2015). Mammographic image analysis based on adaptive morphological fuzzy logic CAD system. Int J Biomed Eng Technol.

[18] Dai S, Ke L, Zhai R, Dong J (2016). Lung segmentation method based on 3D region growing method and improved convex hull algorithm. J Electron Inf Technol.

[19] Toz G, Erdoğmuş P (2021). A novel hybrid image segmentation method for detection of suspicious regions in mammograms based on adaptive multi-thresholding (HCOW). IEEE Access.

[20] Ma J, Song Y, Tian X, Hua Y, Zhang R, Wu J (2020). Survey on deep learning for pulmonary medical imaging. Front Med.

[21] F G, ML D, J Q. Classification of lung nodules by ensemble learning based on Bootstrap-Heterogeneous SVM. J Tianjin Univ (Sci Technol). 2017;3:321–7.

[22] Jia AJ, Liu BJ, Gu CY. Computer-aided diagnosis of pulmonary nodules on CT scan images. In: 2018 10th Int Conf Model Identif Control Icmic. 2018;00:1–6.

[23] Monkam P, Qi S, Ma H, Gao W, Yao Y, Qian W (2019). Detection and classification of pulmonary nodules using convolutional neural networks: a survey. IEEE Access.

[24] Dodia S, Annappa B, Mahesh PA (2022). Recent advancements in deep learning based lung cancer detection: a systematic review. Eng Appl Artif Intel.

[25] Tse ZTH, Chen Y, Hovet S, Ren H, Cleary K, Xu S (2018). Soft robotics in medical applications. J Med Robot Res.

[26] Burgner-Kahrs J, Rucker DC, Choset H (2015). Continuum robots for medical applications: a survey. IEEE Trans Robot.

[27] Ishii H, Kamegawa T, Kitamura H, Matsuno T, Hiraki T, Gofuku A. Development of a prototype of puncturing robot for CT-guided intervention. In: 2016 IEEE 11th Conf Industrial Electron Appl ICIEA. 2016;1020–5.

[28] Hungr N, Bricault I, Cinquin P, Fouard C (2015). Design and validation of a CT- and MRI-guided robot for percutaneous needle procedures. IEEE Trans Robot.

[29] Wang L, Hu T (2020). The design of a dual channel synchronous control system based on a new percutaneous puncture surgical robot. Multimed Tools Appl.

[30] Scali M, Breedveld P, Dodou D (2019). Experimental evaluation of a self-propelling bio-inspired needle in single- and multi-layered phantoms. Sci Rep-UK.

[31] Li P, Jiang S, Liang D, Yang Z, Yu Y, Wang W (2017). Modeling of path planning and needle steering with path tracking in anatomical soft tissues for minimally invasive surgery. Med Eng Phys.

[32] Abayazid M, Moreira P, Shahriari N, Patil S, Alterovitz R, Misra S (2015). Ultrasound-guided three-dimensional needle steering in biological tissue with curved surfaces. Med Eng Phys.

[33] Huang D, Tang P, Wang Y, Li H, Tang W, Ding Y (2018). Computer-assisted path planning for minimally invasive vascular surgery. Chin J Electron.

[34] Yang C, Xie Y, Liu S, Sun D (2018). Force modeling, identification, and feedback control of robot-assisted needle insertion: a survey of the literature. Sensors.

[35] Xiong J, Li X, Gan Y, Xia Z (2015). Path planning for flexible needle insertion system based on improved rapidly-exploring random tree algorithm. IEEE Int Conf Inf Autom.

[36] Lee J, Wang J, Park W (2018). Efficient mechanism design and systematic operation planning for tube-wire flexible needles. J Mech Robot.

[37] Fauser J, Sakas G, Mukhopadhyay A (2018). Planning nonlinear access paths for temporal bone surgery. Int J Comput Ass Radiol Surg.

[38] Zhang R, Wu S, Wu W, Gao H, Zhou Z (2019). Computer-assisted needle trajectory planning and mathematical modeling for liver tumor thermal ablation: a review. Math Biosci Eng.

[39] Setio AAA, Ciompi F, Litjens G, Gerke P, Jacobs C, van Riel SJ (2016). Pulmonary nodule detection in CT images: false positive reduction using multi-view convolutional networks. IEEE Trans Med Imaging.

[40] Armato SG, McLennan G, Bidaut L, McNitt-Gray MF, Meyer CR, Reeves AP (2011). The Lung Image Database Consortium (LIDC) and Image Database Resource Initiative (IDRI): a completed reference database of lung nodules on CT scans. Med Phys.

[41] Meng W, Wang Y (2018). Comprehensive analyses of the elasto-plastic oblique contact-impact with vibration response. Proc Inst Mech Eng Part K J Multi-body Dyn.

[42] Wang Y, Fu Z (2019). Analytical study of babbitt/steel composite structural bars in oblique contact-impact with a solid flat surface. Mech Sci.

[43] Feng H, Xu G, Han Y, Liu Y (2017). A lane line segmentation algorithm based on adaptive threshold and connected domain theory. Ninth Int Conf Graph Image Process ICGIP.

[44] Santamaría-Pang A, Hernandez-Herrera P, Papadakis M, Saggau P, Kakadiaris IA (2015). Automatic morphological reconstruction of neurons from multiphoton and confocal microscopy images using 3D tubular models. Neuroinformatics.

[45] Legland D, Arganda-Carreras I, Andrey P (2016). MorphoLibJ: integrated library and plugins for mathematical morphology with ImageJ. Bioinformatics.

[46] Soltaninejad S, Cheng I, Basu A (2016). Robust lung segmentation combining adaptive concave hulls with active contours. IEEE Int Conf Syst Man Cybern SMC.

[47] Ming JTC, Noor NM, Rijal OM, Kassim RM, Yunus A (2014). Enhanced automatic lung segmentation using graph cut for interstitial lung disease. IEEE Conf Biomed Eng Sci IECBES.

[48] Han F, Wang H, Zhang G, Han H, Song B, Li L (2015). Texture feature analysis for computer-aided diagnosis on pulmonary nodules. J Digit Imaging.

[49] Wang Z, Xin J, Sun P, Lin Z, Yao Y, Gao X (2018). Improved lung nodule diagnosis accuracy using lung CT images with uncertain class. Comput Methods Progams Biomed.

[50] Cline HE, Dumoulin CL, Hart HR, Lorensen WE, Ludke S (1987). 3D reconstruction of the brain from magnetic resonance images using a connectivity algorithm. Magn Reson Imaging.

[51] Li X, Wang X, Dai Y, Zhang P (2015). Supervised recursive segmentation of volumetric CT images for 3D reconstruction of lung and vessel tree. Comput Methods Program Biomed.

[52] Newman TS, Yi H (2006). A survey of the marching cubes algorithm. Comput Graph.

[53] Jian WZ, Zhuang FU, Chenzhuo LU, Yanna Z, Rongli X, Jun Z, Zhuang F, Chenzhuo L, Yanna Z, Rongli X, Jian ZJ (2021). Research on supersonic thyroid piercing three dimensional navigation based on three dimensional reconstruction technology. Mach Electron.

[54] Zhang TF, Fu Z, Wang Y, Shi WY, Chen GB, Fei J (2020). Lesion positioning method of a CT-guided surgical robotic system for minimally invasive percutaneous lung. Int J Med Robot.

[55] Messay T, Hardie RC, Rogers SK (2010). A new computationally efficient CAD system for pulmonary nodule detection in CT imagery. Med Image Anal.

[56] Yi X, Walia E, Babyn P (2019). Generative adversarial network in medical imaging: a review. Med Image Anal.

[57] Zhu Y, Fu Z, Fei J. An image augmentation method using convolutional network for thyroid nodule classification by transfer learning. In: 2017 3rd IEEE int conf comput commun ICCC. 2017:1819–23.

[58] Ji Z, Wang G, Chen B, Zhang Y, Zhang L, Gao F (2018). Clinical application of planar puncture template-assisted computed tomography-guided percutaneous biopsy for small pulmonary nodules. J Canc Res Ther.

